# Underrated aspects of a true Mediterranean diet: understanding traditional features for worldwide application of a “Planeterranean” diet

**DOI:** 10.1186/s12967-024-05095-w

**Published:** 2024-03-21

**Authors:** Justyna Godos, Francesca Scazzina, Corrado Paternò Castello, Francesca Giampieri, José L. Quiles, Mercedes Briones Urbano, Maurizio Battino, Fabio Galvano, Licia Iacoviello, Giovanni de Gaetano, Marialaura Bonaccio, Giuseppe Grosso

**Affiliations:** 1 Department of Biomedical and Biotechnological Sciences, University of Catania, Catania, Italy; 2 Department of Food and Drug, University of Parma, Parma, Italy; 3 Boniviri Società Benefit Certified BCorp, Catania, Italy; 4 Department of Clinical Sciences, Università Politecnica Delle Marche, Ancona, Italy; 5 Research Group on Food, Nutritional Biochemistry and Health, Universidad Europea del Atlántico, Isabel Torres 21, 39011 Santander, Spain; 6 Department of Physiology, Institute of Nutrition and Food Technology “José Mataix”, Biomedical Research Center, University of Granada, Avda del Conocimiento S/N, Parque Tecnologico de La Salud, Armilla, 18016 Granada, Spain; 7 Research and Development Functional Food Centre (CIDAF), Health Science Technological Park, Avenida del Conocimiento 37, 18016 Granada, Spain; 8 Universidad Internacional Iberoamericana, Campeche, 24560 México; 9 Universidad Internacional Iberoamericana, Arecibo, PR 00613 USA; 10 International Joint Research Laboratory of Intelligent Agriculture and Agri-Products Processing, Jiangsu University, Zhenjiang, Jiangsu People’s Republic of China; 11 Department of Epidemiology and Prevention, IRCCS Neuromed, Pozzilli, Italy; 12 Libera Università Mediterranea (LUM) “Giuseppe Degennaro”, Casamassima (Bari), Italy; 13 Center for Human Nutrition and Mediterranean Foods (NUTREA), University of Catania, Catania, Italy

**Keywords:** Mediterranean diet, Plant-based, Sustainability, Planetary diet, Grains, Spices, Olive oil, Legumes, Fish, Herbs

## Abstract

Over the last decades, the Mediterranean diet gained enormous scientific, social, and commercial attention due to proven positive effects on health and undeniable taste that facilitated a widespread popularity. Researchers have investigated the role of Mediterranean-type dietary patterns on human health all around the world, reporting consistent findings concerning its benefits. However, what does truly define the Mediterranean diet? The myriad of dietary scores synthesizes the nutritional content of a Mediterranean-type diet, but a variety of aspects are generally unexplored when studying the adherence to this dietary pattern. Among dietary factors, the main characteristics of the Mediterranean diet, such as consumption of fruit and vegetables, olive oil, and cereals should be accompanied by other underrated features, such as the following: (i) specific reference to whole-grain consumption; (ii) considering the consumption of legumes, nuts, seeds, herbs and spices often untested when exploring the adherence to the Mediterranean diet; (iii) consumption of eggs and dairy products as common foods consumed in the Mediterranean region (irrespectively of the modern demonization of dietary fat intake). Another main feature of the Mediterranean diet includes (red) wine consumption, but more general patterns of alcohol intake are generally unmeasured, lacking specificity concerning the drinking occasion and intensity (i.e., alcohol drinking during meals). Among other underrated aspects, cooking methods are rather simple and yet extremely varied. Several underrated aspects are related to the quality of food consumed when the Mediterranean diet was first investigated: foods are locally produced, minimally processed, and preserved with more natural methods (i.e., fermentation), strongly connected with the territory with limited and controlled impact on the environment. Dietary habits are also associated with lifestyle behaviors, such as sleeping patterns, and social and cultural values, favoring commensality and frugality. In conclusion, it is rather reductive to consider the Mediterranean diet as just a pattern of food groups to be consumed decontextualized from the social and geographical background of Mediterranean culture. While the methodologies to study the Mediterranean diet have demonstrated to be useful up to date, a more holistic approach should be considered in future studies by considering the aforementioned underrated features and values to be potentially applied globally through the concept of a “Planeterranean” diet.

## Introduction

The Mediterranean diet has been widely investigated over the last decades as a virtuous dietary pattern able to match benefits for human health and culinary palatability [1]. In 2010 the Mediterranean diet was recognized as an Intangible Cultural Heritage of Humanity by UNESCO, followed by an active discussion on how to preserve and avoid contamination of such a characteristic cultural lifestyle [2]. There are currently thousands of scientific articles investigating the health and environmental impact of this dietary pattern, either in the Mediterranean area or testing its application in populations living all around the world [3]. While evidence of its health benefits has built up over the decades, important changes have been reached in the definition and understanding of what the Mediterranean diet would have meant in the past and what messages should be projected in the future at global level [4]. Several studies recorded a substantial change in food available in Mediterranean and non-Mediterranean regions [5], suggesting an abandonment of traditional dietary patterns in the former, while a higher adherence to the Mediterranean diet in the latter [6, 7]. The growing number of studies conducted in non-Mediterranean countries, often rigidly applying methodological rules to detect the adherence to the Mediterranean diet in completely different scenarios, may have denatured and not entirely captured the true meaning of adopting a traditional dietary pattern that brings with it the millennial old cultural heritage of various populations and cultures united by living in a close geographical area [8]. Dietary habits are not merely the result of an act to support survival but rather considered a social and cultural phenomenon [9]. In this context, the UNESCO Chair of “Health Education and Sustainable Development” aims to rediscover and define the main features of traditional dietary patterns all around the world proposing a “*Planeterranean*” approach to promote diet, culture, and health [10]. Several factors are embedded with dietary habits, yet they are not taken into account for their health effects while being part of the Mediterranean diet [11–13]. The aim of this review was to provide an overview of historical aspects of the Mediterranean diet and to examine current evidence for better identification and characterization in the modern world, considering important and often underrated aspects.

## History of the Mediterranean diet: the original features

There is no univocal definition for the Mediterranean diet. Although many concepts have been associated with such dietary pattern as will be then discussed in the present article, a generic consideration of the Mediterranean diet relies on the main features characterizing the dietary habits of individuals living in the Mediterranean area, including the Southern European territories, the northern part of Africa, and some Middle-Eastern countries [14]. The geographical location of the Mediterranean populations, along with a favorable climate, was responsible for some specific features that made the diet followed by people living in the Mediterranean area unique. There is consistent documentation of consumption of certain foods in ancient times: although it is not possible to have certainty concerning the frequency (i.e., daily intake) and common use among the general population (in contrast to limited consumption only in certain social classes), there are some proofs from prehistoric ages until more recent times that certain foods were, in fact, peculiarly included in the common dietary habits in Mediterranean populations compared to those from other geographical regions [15, 16].

Starting from the agricultural revolution (the prehistoric transition from hunting and gathering to settled agriculture more than 10,000 years BC) there has been a substantial differentiation in human behaviors, and consequent cultural evolution across civilizations [17]. While some ancestors kept living in nomadic state following the availability of hunted/gathered food, others settled down and developed technological skills, accompanied by social and cultural development [18, 19]. The area where the first populations settled down was the Fertile Crescent, the Middle East region spanning from modern-day Iraq, Syria, Lebanon, Israel, Palestine, and Jordan, and partially Turkey and Iran. Starting from the riversides of Tigris, Euphrates, and the Jordan River, early civilizations migrated toward the North-Western Mediterranean area and further settled in a more favorable environment [20]. The Mediterranean area provided a mild climate ideal to nurture biodiversity. A peculiar feature characterizing the agriculture of this area was the great variety of fruit and vegetables that various civilizations were able to cultivate and spread all over the territory facing the Mediterranean Sea. There are plenty of characteristic fruits and vegetables that gathered popularity among the civilizations that have taken place in this area. Among fruits, early domestication of pomegranates, figs, dates, and later pears and apples, citrus fruits, and apricots were quite spread all over the Mediterranean area [21]. Among vegetables, the Arab and Islamic influence introduced several products, such as aubergines, spinach, lettuce, asparagus, carrots, cucumbers, melons, and watermelons [22]. Nowadays, many more plant foods are considered characteristic of this area, although they have been only in relatively recent times imported from other geographical areas and just later included in Mediterranean cuisine, such as cherry tomatoes and artichokes.

Aside from this long and constantly evolving history concerning fruit and vegetables (for instance, now avocados and mangos are being introduced in Southern Italy), back in the early times, the consumption of three main food groups truly differentiated the Mediterranean Region against the other territories: olives, grapes, and cereals [23].

### Olives

The most recent common ancestor of various olive subspecies has been estimated to have occurred about 6 million years ago but only relatively recently domesticated in the region of Persia and Mesopotamia [24]. The introduction of the olive tree (*Olea europaea *L. subsp*. europaea* var*. europaea*) in the Mediterranean Region seems to depend on Phoenicians: it has been estimated that this culture spread olives in the various regions of the Mediterranean in the first millennium BC [25]. However, it was only with the Greek civilization that olives assumed some sort of distinctive “identity” of the Greek empire and the whole Mediterranean region [26]. Olive products represented a distinctive sign of wealth and a source of power in ancient Greece, the value of which was later recognized also by the Roman Empire and its spread in southern Italian and Spanish areas [27].

### Grapes

Following the development of pottery during the later Neolithic, early discoveries dated 11,000 BC provide evidence of certain fermenting practices of the fermenting strains of this wild *Vitis vinifera* subsp*. sylvestris*, the ancestor of the modern common wine grape [28]. Similarly to olives, grapes were later domesticated over 6000 years ago in the same geographical area (between Persia and the Black Sea), soon after used for winemaking first in Greece and Italy, and only centuries later in Spain and France [29]. The development of technology for wine production and transportation accompanied by a growing demand throughout the region led to the promotion of viticulture as a major driver for economic growth across the Mediterranean area [30]. Part of the success of such a product was the effect on consciousness, which was attributed to religious practices in all civilizations inhabiting the Mediterranean area, from the superstition surrounding wine-drinking in the Egyptian culture due to its resemblance with blood, to the tribute offered from the Greek/Roman to Dyonisius and Baccus, and then to the Jewish practice and later Christian eucharist [31, 32].

### Cereals

When settled farming emerged, the growing groups of people becoming more organized over time were able to modify the natural vegetation toward cultivation of newly domesticated plants as crops [33]. In this context, a variety of cereals (i.e., primarily wheat, but also barley and spelled) were introduced in the Mediterranean area and consumed alongside pulses including pea (*Pisum sativum*), lentil (*Lens culinaris*), broad bean (*Vicia faba*), and chickpea (*Cicer arietinum*) by populations, such as the Phoenicians and the Carthaginians [34]. Cereals were consumed in various forms, such as porridge or bread, but also used for sweets (i.e., biscuit-type cookies); other civilizations, such as Greek and Roman ones, considered bread as a key component of their diets while later on, more complex forms of grains included pasta and wheat middlings (“semolina”) products, such as *couscous* and *bulgur* [35].

## The Mediterranean diet in science: the main features in modern times

Contrary to the common belief that Ancel Keys was the very first scientist digging into Mediterranean dietary features, one of the first scientific attempts to better understand and characterize the eating habits of individuals living in the Mediterranean area was a survey conducted in Crete during the post World War II period [36]. The survey coordinated by Allbaugh and colleagues revealed substantial differences between the diet commonly consumed in Crete compared to the American one: as today we would expect, there was a substantial difference in daily energy intake from plant and animal foods [36]. Curiously, the delay in the industrialization and development of disadvantaged areas (such as Crete), which allowed maintenance of traditional dietary habits as opposed to the modernization of the US food culture, was paradoxically seen as a limitation to the diet quality. The study finally concluded to recommend an increased consumption of animal products.

The most known study of the Mediterranean dietary habits (in Southern Italy, specifically) was then the following work of Keys and its “Seven Countries Study” [37]. Although some hypotheses have then been demonstrated to be rather overestimated (i.e., the lipid hypothesis and the detrimental role of fats in the diet), Keys was the first to suggest that the dietary habits of people living in the Mediterranean area could be responsible for the substantial lower rates of cardiovascular diseases [38]. The documents identified the features of a traditional Mediterranean diet, with emphasis on plant-based foods, but also on the consumption of legumes and cereals in the form of pasta and bread, as well as limited intake of meat, fish, and dairy products [39].

The Mediterranean diet has been continuously studied over the decades by several research groups. Earlier studies in Greece worked over an index score helping to assess the level of adherence to the Mediterranean diet and its association with mortality risk [40]. Another landmark study from Spain, the PREDIMED (*Prevención con Dieta Mediterránea*) trial, further confirmed with an experimental design that higher adherence to this dietary pattern would reduce cardiovascular risk [41]. A myriad of other studies have been conducted to test the relationship between the Mediterranean diet and health outcomes, overall concluding substantial benefits of adopting a Mediterranean diet toward cardio-metabolic and neurodegenerative diseases and certain cancers [42].

**Fig. 1:**
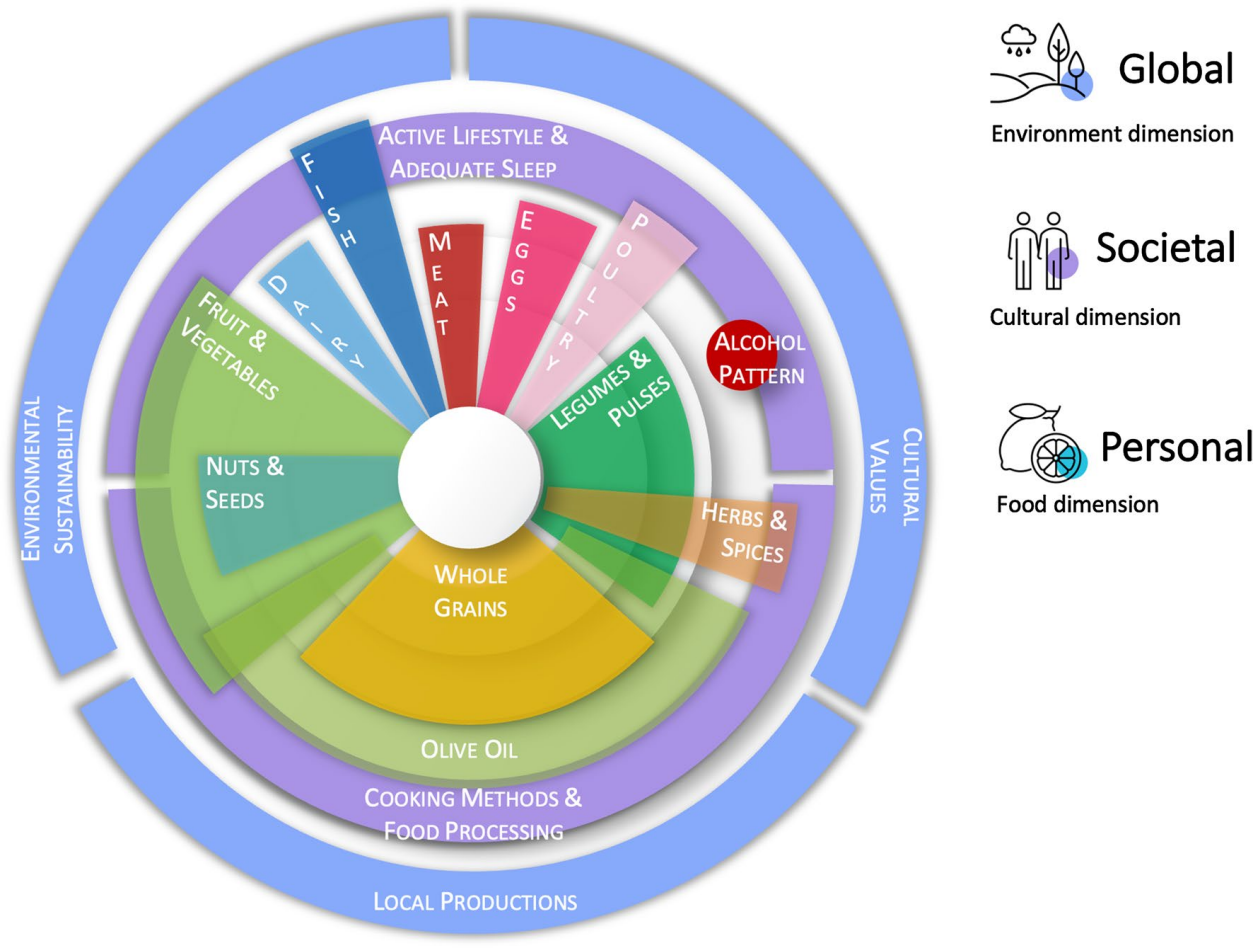
.

To perform all such studies, a variety of indexes have been developed and used to detect and quantify the level of adherence to the Mediterranean diet by focusing on those features that would most represent this dietary pattern [43]. These indexes generally took into account daily/weekly portions or quantiles of intake to assign the scores indicating the level of adherence [44]. The use of dietary scores and indexes is convenient for the wide applicability in epidemiological studies and easy to perform given the relatively limited number of food groups generally included. While previous studies described in detail the features of the many scores [45], some key components that are consistently evaluated and the rationale for their benefits on health are listed below (Fig. 1).

### Fruit and vegetables

The Mediterranean diet is primarily a plant-based dietary pattern. The consumption of several portions of fruits and vegetables daily is a constant characteristic of all dietary indexes evaluating the level of adherence to the Mediterranean diet.

The continuous introduction of vegetable varieties over the centuries has led to a wide diversity of products including green leafy vegetables and lettuce, tomatoes, carrots, cucumbers, and also less common, yet largely consumed vegetables in certain Mediterranean areas, such as artichokes [46]. Similarly, while widespread fruits such as citrus fruits, some berry fruits (mostly strawberries and cherries), medlars and plums, apples, and pears are commonly available, other specific varieties, such as prickly pears and pomegranates are more characteristic of specific regions within the Mediterranean countries [46, 47].

Most simple sugars are supposed to come from fruits. Fruits and vegetables play an important role to supply fiber, moreover are sources of minerals and vitamins, needed to avoid micronutrient deficiencies, as well as the main source of phytochemical compounds, such as (poly)phenols, that have been reported in several comprehensive summary of evidence to potentially play an important role for human health through the prevention of chronic diseases, including certain types of cancer [48, 49], cardiovascular [50, 51] and neurodegenerative diseases [52], and mortality [53]. While tackling malnutrition issues, some vitamins and phytochemicals may show, to a various extent, antioxidant and anti-inflammatory properties, which play a role in the prevention of non-communicable diseases [54]. Nonetheless, recent evidence shows that plant-based fiber-rich foods also affect quality and variety of gut microbiota, which is currently studied as a potential modulator of the immune system as a determinant of health [55].

### Olive oil

Remarking the main original features characterizing the primary aspects of the early dietary habits in the Mediterranean area, these three components are always (with some exceptions concerning legumes) reported in all dietary indices identifying the main Mediterranean dietary features. Olive oil is one of the main sources of mono-unsaturated fatty acids (MUFAs) in people living in the Mediterranean region [56] and also provides peculiar (poly)phenol compounds (such as, hydroxytyrosol) [57–59]. Dietary richness in MUFAs characterizing high adherence to the Mediterranean diet has been considered a healthy feature, since MUFA may counteract the pro-inflammatory activity of saturated fats and exert anti-inflammatory effects through a variety of mechanisms [60]. Olive oil consumed in the Mediterranean countries is generally an extra-virgin olive oil, which is also rich in other compounds, such as triterpenes (i.e., squalene), phytosterols (i.e., b-sitosterol), tocopherols, and (poly)phenols (oleuropein and hydroxytyrosol) reported to exert potential effects on human health [61]. Notably, some studies conducted outside the Mediterranean area used a nutrient-based approach to investigate the potential effects of olive oil on health by testing for the ratio between MUFA and total fats (rather than consumption of olive oil per se): although such an approach would allow the application of the index in populations with known scarce consumption of olive oil, the results showed that an important source of MUFA outside the Mediterranean area may also be represented by meat, thus leading to a substantial validity bias of the scoring system [62]. A comprehensive summary of the literature showed that there is evidence of the association between olive oil consumption and reduced risk of cardiovascular disease (CVD) and type-2 diabetes [63]. Meta-analyses of clinical trials additionally showed that olive oil intake had little effect on the blood lipid profile [64], but significantly improved markers of oxidative stress, such as oxidized low-density lipoprotein (LDL) and malondialdehyde levels [65]. Olive oil consumption has also been associated with a lower likelihood of having cancer, including breast, upper aerodigestive, gastrointestinal, and urinary tract cancers [66].

### Wine

Since one of the characteristic crops of the Mediterranean area was vine, the peculiarity explored in Keys’ study on Southern Italian people was moderate daily wine drinking, especially red wine. The peculiarity of (red) wine choice in moderation seemed to have gained interest in researchers, who started exploring its potential benefits. There is consistent evidence that moderate alcohol consumption (especially wine) would be associated with lower risk of cardiovascular diseases, thus adding up to the overall positive impact of the whole Mediterranean diet on the cardiovascular system [67]. Alcohol per se may reduce plasma viscosity through reduction of fibrinogen concentration and platelet aggregation, as well as increase high-density lipoprotein cholesterol [68, 69]. Moreover, red wine is rich in certain (poly)phenols, such as resveratrol and anthocyanins, which are among the most studied (and provided with the best evidence, albeit with some limitations) for their positive effects on endothelial and vascular health [70].

### Whole grains

Whole grain cereals are supposed to provide the largest daily energy share to individuals adhering to the Mediterranean diet. During the early times of Mediterranean history, wheat (*Triticum aestivum*, *Triticum durum*) represents one of the cornerstone characteristics of the Mediterranean diet [71]. Barley (*Hordeum vulgare*) cultivation is dated about 10,000 years ago in the Fertile Crescent and it was probably one of the most important grains of ancient civilizations [72]. Other ancient hulled wheat grains were einkorn (*Tricum monococcum*), emmer (*Triticum dicoccum*), and spelt (*Triticum spelta*), while products more peculiar of Eastern countries include *freekeh* (also known as *farik*, a wheat harvested when green and then roasted) and *bulgur* (cracked and parboiled whole wheat pieces) [73]. Nowadays, cereals are widely consumed in all Mediterranean countries in a large variety of forms and recipes. Barley is double baked into hard crunchy bread called *rusks*, or *paximadia*, generally softened up with dressings (including cheeses) and dips, or as the base of *Dakos* (tomatoes, feta cheese, oregano, and extra-virgin olive oil) [74]. *Bulgur* and couscous (Durum wheat *semolina*) are used in Eastern Mediterranean countries as main cereals in salads (i.e., the Turkish *kisir*) with vegetables and sauces [75, 76]. In Italy, cereals are mainly consumed as bread and *pasta* of various shapes and compositions or *risotto* (rice per se with a variety of sauces and preparations); in Northern Italy, whole grain corn flour can be used to cook *polenta*, a creamy versatile preparation that can be baked, boiled, or fried into a harder cake [77]. Rice is also widely consumed in Spain as the main cereal ingredient of *paella* [78]. Other types of flatbreads more common in Eastern Mediterranean countries include *aish baladi*, *pita*, or *lavash* [79].

As one of the first characteristics of the Mediterranean area, cereals are constantly included in the majority of dietary indexes. However, a limitation worth mentioning is the inclusion of cereals in Mediterranean dietary indexes with no consideration for the level of processing. Looking back at history, the cereals and derived products (bread, pasta, etc.) commonly consumed were whole grain cereals: the process of refining only occurred over the last 50 years until nowadays most available products are made of refined cereals (or flour), impoverished of the germ and bran [80]. Whole grain cereals are among the few food groups for which there is convincing evidence of their benefits for a variety of outcomes, including metabolic disorders (type-2 diabetes), CVD, and cancer (colorectal) [81]. Intervention trials specifically demonstrated certain effects in improving blood glucose and cholesterol [82–84]. The benefits related to their consumption rely on richness in both soluble and insoluble functional dietary fiber (roughly 10 g per 100 g of product) including resistant starch, β-glucan, cellulose, arabinoxylan, xyloglucan, and fructan [85]. Whole grains are also rich in minerals (selenium, zinc, copper, and magnesium), vitamins (vitamin E), carotenoids, and (poly)phenols in the germ [86, 87]. Thus, the loss of those important components after refining in favor of a sweeter taste substantially lowers the overall quality of such a major feature of a traditional Mediterranean diet.

### Legumes and pulses

Alongside whole grain cereals, also consumption of legumes is considered a consistent feature characterizing the Mediterranean diet. Legumes include the seeds of the plant Leguminose and are referred to as pulses commonly consumed throughout history due to their nutritious and filling characteristics [88]. The most common legumes consumed in the Mediterranean area include faba beans (*Vicia faba* L.), chickpeas (*Cicer arietinum*), lupins (*Lupinus albus*), and lentils (*Lens culinaris*) [89]. The first consumption of legumes probably dates back to around 10,000 years ago in the Middle East and Crescent Fertile and then spread to the Mediterranean area a while later [90]. Analyzes reconstructing the diet of Greek populations reveal the importance of legumes as the main staple food providing carbohydrates and proteins during the sixth and fifteenth centuries [91]. In Roman times, or the medieval period, legumes were mixed with other grains that produced dark types of bread, leaving white bread for higher social classes individuals [92]. During the ‘60, legumes were part of the traditional Mediterranean cuisine due to their relatively inexpensive price, the high nutritional values, and the satiating properties [93]. However, their convenience has been contrasted by other alternatives, either meat-based among higher socioeconomic populations or processed alternatives, leading to a substantial decrease in global legume consumption, especially in Western countries [94]. Nowadays, legumes are consumed in typical dishes, such as hummus (a nutrient-dense dip or spread made from cooked, mashed chickpeas, blended with *tahini*, olive oil, lemon juice, and spices) or used in stews and *minestrone* to enrich the mixture of cereals and vegetables [95].

Curiously, not all indexes evaluating the level of adherence to the Mediterranean diet include legumes as a major feature, thus missing the chance to measure whether such a food group is actually consumed in Mediterranean populations. Legumes are generally low in saturated fats, but they can contain, to a different extent depending on the type of seed, a variable amount of MUFA and poly-unsaturated fatty acids (PUFA) [96]. Together with cereals, legumes constitute an important source of carbohydrates, fiber, and protein: while being rich in lysine, legumes may not sufficiently provide the whole pool of essential amino acids because poor in sulfur-containing amino acids (methionine and cysteine) and tryptophan; however, they are complementary with whole grain cereals, with which they would provide complex dishes with a good quality protein [97]. Legumes are also rich in vitamins (such as vitamin C, thiamin, riboflavin, niacin, pantothenic acid, pyridoxine, biotin, and folate), minerals (including calcium, magnesium, phosphorus, copper, manganese, selenium, iron, zinc), phenolic compounds (including phytoestrogens, such as lignans and isoflavones), and phytosterols [98]. There is suggestive evidence that higher intake of legumes is associated with lower risk of CVD [99] and improvement in insulin sensitivity and blood glucose levels [100]. Other studies showed that legumes would also improve plasmatic levels of LDL cholesterol and triglycerides as well as affect blood pressure and endothelial function [101]. Legumes are often being called out as potentially deleterious for human health due to their content in anti-nutrients, including protease inhibitors, lectins, phytic acid, oxalate, and saponins that may reduce the bioavailability of some nutrients; however, certain procedures, such as soaking, sprouting, or simply cooking (i.e., boiling) seem to improve their digestibility and significantly limit their anti-nutrient properties [102].

### Meat

Another feature consistently present in most indexes is the limited intake of meat. Historically, consumption of meat from sheep, lambs, cattle, and pigs was part of the dietary habits of individuals living in the Mediterranean area [103]. The domestication of animals helping mankind but then being killed provided the rationale for the rituality of the process, often accompanied by the sacrality of religious feasts and social celebrations [104]. During the Roman period, meat consumption grew more common among the upper social classes while the increasing expansion of the Empire toward barbarism raised the importance of such food as the most important source of energy and strength [105]. Based on data starting from the nineteenth century, the overall meat consumption in the Mediterranean area was certainly low, especially when compared to American and British dietary habits [106]. The delay of economic development became some sort of advantage toward the reduction of the risk of non-communicable diseases: while the Westernization of the diet led to excess consumption of meat products as opposed to the complete lack (and the associated hunger and malnutrition issues), moderation resulted as the key for the benefits observed in the modern Mediterranean diet [107]. Nowadays, all types of meat are consumed in southern European countries, while preference for lamb over pork is observed in Muslim Mediterranean countries; both fast (roasted) and long cooking methods (stews and “pulled”) with olive oil and spices are common in all cultures, with rising trends of consumption over time [108].

Meat is a nutritious source of high-quality proteins and a large share of vitamins, some of which are essentially provided almost entirely by animal products (i.e., some group B vitamins). In spite of that, current trends show an increasing consumption of meat in developed countries while evidence suggests that extreme intake (compared to lower) could actually increase the risk of cardiovascular disease and certain cancers (i.e., colorectal cancer) [109]. A variety of mechanisms (not unequivocally identified) related to meat per se and its processing and cooking methods have been suggested, including the presence of heme iron (which can induce endogenous production of N-nitroso compounds [110], gut-liver production of cardiotoxic trimethylamine *N*-oxide (TMAO), and formation of meat mutagens, such as heterocyclic amines (HCA) when cooked in high temperatures, while processed meat contains additives such as nitrates and nitrites, precursors of N-nitroso compounds [111]. However, most indexes fail to distinguish meat consumption by level of processing, which is in fact reported to be an important discriminating variable to affect the impact of meat on human health [109]. Excess intake of processed meat (including meat undergoing processes such as salting, smoking, curing, etc.) has convincingly been demonstrated to be associated with the aforementioned detrimental outcomes, while the evidence on the negative effects of red and white unprocessed meat is weaker, if not null, depending on the outcome investigated [109]. Certainly, the “dose” makes the difference, but the dietary indexes currently used to assess the level of adherence to the Mediterranean diet do not take into account such recommended intakes, but rather promote increased scoring (meaning higher adherence to the Mediterranean diet) for lower intake of meat. While conceptually correct, some reports suggested that plant-based dietary patterns do not always reflect healthiness [112]. Other aspects, such as the level of processing and cooking methods are also generally not considered, yet equally important to be considered when exploring meat consumption, also in the context of a Mediterranean diet.

### Fish

Fish is a product generally consumed since early times by the populations living in the coastal areas of the Mediterranean Region [113]. During the Greek period, specific markets dedicated to seafood supplied all social classes with cheaper (i.e., anchovies and sardines) or more expensive (larger fishes and shellfish) products; while the main attention for prestigious courses interested meat, the subsequent fasting periods or forbidden meat consumption kept fish as a secondary, yet common preference in the following times [114]. More sophisticated preparation methods followed during the Roman period and later, aiming to prolong preservation and increase palatability [115]. Thus, depending on the possibility, consuming fish from the Mediterranean Sea has historically been an “involuntary” advantage of populations living in this region [116]. Ancient civilizations consumed fish simply roasted, salted, or blended with wild herbs [117]. South-Eastern Mediterranean countries consumed less fish compared to European Mediterranean ones, with such differences still observed nowadays [118].

As commented on meat consumption, lack of information concerning type and size of fish, as well as level of processing limits the reliability of the majority of indexes to assess the level of adherence to the Mediterranean diet. Fish is one of the most important sources of PUFAs, such as eicosapentaenoic acid (EPA) and docosahexaenoic acid (DHA) omega-3 fatty acids, which have been reported to potentially play an important role as anti-inflammatory agents (because precursors of molecules involved in the resolving phase of the inflammatory process) and consequently associated with reduced risk of cardiovascular diseases, depression, and certain cancers (liver and pancreas) [119, 120]. Omega-3 PUFAs can be assumed also from plant sources (i.e., seeds) as alpha-linolenic acid (ALA); however, the active form necessary to the human body is DHA, and the enzymatic processes ALA needs to undergo do not allow a sufficient amount to be transformed to supply the body’s needs [121]. Concerns regarding the impact of the Mediterranean Sea pollution alarmed the consumers on the rise in heavy metal content (i.e., mercury) in fish [122, 123]. However, the content in small-size fish seems not to overcome the benefits of omega-3, thus not affecting current guidelines concerning fish consumption [124]. In contrast, preserved fish may result in high content of sodium and related health threats to be considered [125]. In fact, while individuals living in coastal Mediterranean regions are more likely to consume fresh fish (with the associated benefits), most scores fail to address which type of fish is consumed in non-Mediterranean countries (i.e., salted fish).

## Underrated aspects of the Mediterranean diet

The aforementioned features representing the fundamentals of the modern Mediterranean diets lay the ground directly to the main dietary habits occurring in the Mediterranean area over the millennia. However, some limitations are intrinsic to the methodology used. For instance, dietary scores and indexes do not assess the total daily calorie intake and, most importantly, the proportion of macronutrients (ideally assessed to be approximately 50% daily energy from carbohydrates, 30% from fats, and 20% from proteins). Moreover, the definition a priori of food groups to be considered, lack of tolerance limits, and attribution of equal weight to all components (independent of their proportion in the overall diet) may constitute additional sources of bias. Other limitations include the use of quantiles to stratify the population by different levels of adherence to the Mediterranean diet when large score variations across countries can be observed (the lowest quantile of exposure in a Mediterranean population may potentially display higher scores than the highest quantile in a non-Mediterranean population); alternatively, the use of cut-off points may be an arbitrary choice, limiting the overall comparability of results across studies as well.

Aside from methodological limitations, other aspects related to this dietary pattern are generally poorly emphasized or rather not taken into consideration. Current classification through dietary scores and indexes do not and cannot fully capture the cultural, behavioral, and culinary features characterizing the Mediterranean dietary pattern adopted by individuals living in the Mediterranean area, from its historical beginning to the southern Italian farmers in the ‘60 s. If from one side the adherence scores used by the vast majority of the scientific studies are designed to include the main nutritional features supposed to play a role on human health, other aspects are nearly never taken into account. Besides, some aspects considered secondary or marginally relevant appearing at the middle of the Mediterranean diet pyramid, are nowadays studied with a more holistic approach and considered underrated (Fig. 1). While research on the Mediterranean diet has grown to the point of leaving no doubts about its benefits, further discussion is encouraged by considering future in-depth studies on the following matters.

### Eggs

Egg consumption is generally not included in dietary indexes used to assess the level of adherence to the Mediterranean diet. One can hypothesize that at the time of the first discoveries on the relation between diet and human health, with the very first investigations on the Mediterranean diet, eggs have been in the spotlight (since then till up to now) as a major source of cholesterol and, based on the lipid hypothesis (promoted by Ancel Keys himself), considered a possible risk factor for cardiovascular diseases. Given the negative perception generated on fat-rich foods, the inclusion of eggs as a part of the Mediterranean diet would have caused confusion, if not conflict, in the scientific community. However, eggs are a nutritious inexpensive source of proteins, minerals, and vitamins, very likely to be commonly produced and consumed in the countryside of southern Italian villages where the first scientific studies on the Mediterranean diet have been based. However, after all these years and so many published articles, egg consumption has never been considered in the context of the Mediterranean diet and evaluated for its association with health. In the context of the Mediterranean diet, eggs are generally consumed boiled, medium-cooked, sometimes opened on a hot plate or just slightly fried with half a spoon of extra-virgin olive oil (no butter). More complex recipes may include *shakshuka*, a recipe commonly consumed in North Africa and Middle Eastern countries consisting of poached eggs alongside tomato and bell pepper sauce. Overall, egg consumption has never been taken into account as a major characteristic of the Mediterranean dietary pattern, but its inclusion would have probably led to different findings whether investigated in contrast to the American/British dietary habits as consumed together with butter, bacon, or sausages. There is comprehensive evidence from the scientific literature showing that there is no relation between egg consumption and detrimental effects on health [126], also with specific regard to cardiovascular disease risk [127]. Current knowledge on cholesterol absorption from food has developed over time providing evidence that dietary cholesterol has only partial (if any, mostly depending on genetic factors) influence on blood circulating levels of LDL-cholesterol (the one actually to be incriminated for being a cardiovascular risk factor), which in fact is mostly dependent on endogenous synthesis (from the liver) [128]. It is noteworthy to underline that some individuals might be more sensitive to the cholesterol content in eggs (the so-called cholesterol “hyper-absorbers”) and could have detrimental effects on CVD risk due to production of TMAO, as already reported for meat products [129]. Aside from these personalized nutrition aspects (which may find their application in the near future in a clinical setting), the general effects of eggs on the global population are rather beneficial. Egg protein is highly digestible and a source of essential amino acids, with a high attainable protein digestibility-corrected amino acid score [130]. Among them, eggs are a dietary source of bioactive peptides, which can exert antioxidant and anti-inflammatory activities [131]. Fats contained in eggs do not only include cholesterol, but also phospholipids, mono- and saturated fatty acids, lutein, lecithin, choline (important for the nervous system), and a variety of vitamins and minerals [132].

### Dairy products

A similar discussion is valid for dairy products as well. Historically, milk and its derivates (butter, yogurt, curd, and buttermilk) have been part of the common diet in the Mediterranean area for at least 9000 years, when herd animals such as sheep, goats, cattle, and camels were domesticated [133]. Over the generations, humans have developed the capacity to digest lactose during the whole lifespan, hence allowing milk consumption with no discomfort or disabling symptoms representing an evolutionary advantage of people who raised livestock [134]. Dairy products involved the inclusion of a variety of milk sources, including cow’s, sheep’s, goat’s, and buffalo’s: such differences in nutrient composition and flavors together with preservation techniques led to a peculiar production of cheeses and derivatives (from yogurt to Italian *ricotta* and *mascarpone*) [135].

Once again, the advice of assuring an adequate consumption of dairy products high in saturated fatty acids would have contrasted with the lipid hypothesis. However, dietary saturated fatty acids only partially affect circulating LDL cholesterol while also increasing high-density lipoprotein (HDL) [136]. Moreover, dairy products are characterized by a variety of saturated fatty acids including long-, medium-, and short-chain fatty acids (in contrast to meat products, which only contain long-chain saturated fatty acids). Both medium- and short-chain fatty acids have been hypothesized to exert health effects related to metabolism improvement and microbiota dysbiosis prevention [137]. Moreover, milk and its derivatives are also rich in other compounds that have demonstrated potential benefits against CVD, such as oligopeptides (which may exert angiotensin I-converting enzyme-inhibitory, antihypertensive effects), minerals (including magnesium and calcium), and vitamin D (reported to interact with receptors in cardiomyocytes, endothelial, and vascular smooth muscle cells) [138].

In line with the aforementioned suggested mechanisms, current comprehensive evidence shows that higher dairy product intake is associated with lower risk of hypertension (compared to low consumption), while studies on CVD risk and mortality reported similar risk estimates, although with some sort of heterogeneity across studies (possibly due to fat content in products and geographical localization of populations investigated) [139]. Interestingly, higher dairy consumption is also convincingly associated with lower risk of colorectal cancer, probably due to potential effects on the gut microbiota and the key role of short-chain fatty acids in regulating gut inflammation and colonocytes cell cycle [140].

### Nuts and seeds

Although nuts (walnuts specifically) were represented in one of the two intervention arms of the PREDIMED trial, the evaluation of nut consumption is not univocally included in the variety of Mediterranean diet adherence scores used in the scientific literature (sometimes merged with fish consumption) [141]. Moreover, the type of nuts within the context of Mediterranean diet scores is explored even more rarely (if ever) [142]. Certain types of nuts, such as almonds, walnuts, and pistachios are native from the Mediterranean region or Middle-Eastern/Western Asian countries and subsequently widely spread in the Mediterranean area thousands of years ago [143]. Almonds found their way into the culture of the Roman empire and the northern countries of Africa through the Arabs, consumed raw or roasted into complex dishes, sauces, oils, and desserts, together with almond milk [143]. Greeks were the first to cultivate walnuts, which were used not only for culinary purposes, but also as medicinal food [143]: however, consumption of walnuts has been dated a longer time before in the area of Persia and Mesopotamia [143]. Also Pistachios had their probable origin in Middle Eastern countries, while being introduced in Italy during the Roman domination and spread from there to other European Mediterranean countries [143].

Together with nuts, some seeds have also been historically consumed in the Mediterranean region. Pine nuts are the seeds of the pine tree that have their origin in the eastern Mediterranean countries: they were used as medicinal foods by ancient Egyptians and then spread across the whole Mediterranean area, with the larger production led by Spain [144]. Flaxseeds are derived from the flax (or linseed) plant, first domesticated in the Fertile Crescent region and later spread in the Eastern Mediterranean areas, Egyptians, Phoenicians, and Romans [145]. Other seeds not originally native from the Mediterranean region, such as pumpkin and sunflower seeds, while first grown in the Americas, have been imported into Europe after the discovery of these lands and later widely introduced in Mediterranean dietary cuisine [146, 147].

The health benefits of nuts and seeds are well documented in the scientific literature [148]. Consistent results are showing an association between nut consumption and lower risk of CVD in a dose-dependent manner, while smaller or unclear associations with risk of stroke and type 2 diabetes have been reported [149]. Moreover, findings from randomized controlled trials administering nuts as intervention arm confirm their potential effects on total and low-density lipoprotein cholesterol [149]. Concerning seeds, only a minority of studies have been conducted on commercial products, such as sesame [150, 151], fenugreek [152], and flaxseeds [153, 154] reporting significant effects of markers of inflammation and CVD risk factors, albeit not with univocal result [155–157]. These energy-rich foods are characterized by richness in essential fatty acids and fiber that may be responsible for their cholesterol-lowering properties [158]. Aside from their favorable saturated-to-MUFA (in hazelnuts, peanuts, and almonds) or PUFA (in walnuts and seeds) ratio, such foods are also rich in various phytochemicals [159]. Among them, (poly)phenols (i.e., phenolic acids) exhibit antioxidant and anti-inflammatory properties [160], and phytosterols (especially pistachios and almonds), which may compete with cholesterol for its absorption and enhance its excretion [161]. Intermediary effects of nuts and seeds consumption have been reported on immune and endothelial health, with reduction of inflammatory biomarkers and improvement of vascular function [162].

### Herbs and spices

Hardly found in any Mediterranean diet adherence score, the consumption of herbs and spices is long documented in the Mediterranean area [163]. There is no univocal definition for herbs and spices, hence often generating confusion and overlapping ideology beyond both groups of products: herbs are plants (whole or parts of them, such as the leaves) used to provide a characteristic flavor to a dish while spices can be considered as any aromatic product derived from plants in the form of powder, seeds, or other pulverized plant parts used to provide seasoning to the food. Several common herbs, including fennel (*Foeniculum vulgare* Mill.), bay laurel (*Laurus nobilis* L.), parsley (*Petroselinum crispum*), rosemary (*Rosmarinus officinalis* L.), and sage (*Salvia officinalis* L.) are native from perennial and/or evergreen plants of the Mediterranean basin (especially southern European countries), while others commonly used and nowadays fully integrated within the Mediterranean culture, such as basil (*Ocium basilicum*), oregano (*Origanum vulgare*), chives (*Allium schoenoprasum* L.), capers (*Capparis spinosa* L.), and garlic (*Allium triquetrum*) were imported from Asian or Indian regions about 2–2500 years ago and only later spread in Southern Europe [164]. In contrast, most spices included in the Mediterranean cousin, such as saffron, cumin, and sumac have their origin in Northern Africa (such as Egypt) and Middle-Eastern countries (today Iran and Syria) dated up to seventh-century BC [165]. The use of herbs and spices represents a crucial aspect of tradition linked to a territory and provides a well-defined geographical localization of the origin of the dishes in the context of the Mediterranean area [166]. Herbs are almost ubiquitously used in Mediterranean dishes in order to provide freshness and peculiar tastes to sofrito or freshly added on top of the ready meal. Saffron is used to flavor a variety of dishes in several Mediterranean regions, from Spain (in *paella*) to Iran (in *khoresh*), passing through France (in *bouillabaisse*) and Italy (in *risotto*). Cumin, aside from its inclusion to flavor cooked dishes (i.e., into *sofrito*), can also be found in several traditional spices blends (such as, the *bahaarat* used to flavor various types of meat), some cheeses (i.e., the Leyden cheese) and some traditional bread in France. Sumac is common in the Arab cuisine added to dishes, such as *hummus* and *tashi*, as well as salads, rice, and kebab. Both herbs and spices are vastly studied in scientific literature for their potential health benefits. Several herbs (such as, Thymus spp., Mentha spp., Salvia spp., Foeniculum vulgare) are shown to exert acute effects on the gastrointestinal system, such as improving digestion by increasing the excretion of digestive enzymes [167]. Although consumed only in small amounts, their inclusion in the common cuisine, consistency of daily intake over time, and variety of products used may support the hypothesis that herbs and spices may play a role in the beneficial effects of Mediterranean diet against non-communicable diseases [168, 169]. Progress in research has unveiled a great number of phytochemical compounds contained in both herbs and spices, which are now recognised as functional foods [170]. Both these groups of products are colorful plant derivatives accounted among the richest sources of (poly)phenols [171]. The main hypothesized mechanisms of action rely on their antioxidant and anti-inflammatory properties [172]. Some compounds have documented activity to counteract metabolic disorders (i.e., the well-known cholesterol-lowering and (modest) blood pressure-lowering effects of garlic). Also a variety of actions against cancer (via inhibition, through various mechanisms, of cancer initiation, promotion, growth, or dissemination) [173, 174] and mental health outcomes (including cognition and improved brain functionality) [175] have been documented. In general, most existing evidence is based on animal in vivo studies, in which selected compounds contained in both herbs and spices are associated with potential health benefits [172]; however, clinical trials are rather scarce and further studies are needed to confirm such mechanisms also in humans.

### Cooking methods

While the indexes of adherence to the Mediterranean diet correctly point out which dietary components may characterize the main features of this dietary pattern, most of them fail to evaluate the cooking methods such food is prepared. The variety of preferences for certain foods in contrast to the common aforementioned main features characterizing the Mediterranean diet is, in fact, mostly emphasized by the large variety of distinct regional cuisines, within the European countries (Greek, Italian, French, and Spanish) and those from the Middle Eastern area (Maghrebi, Levant, and Ottoman). In general, vegetables are commonly consumed raw as part of fresh salads supposed to accompany each meal, rarely dressed with anything more than olive oil and vinegar [176, 177]. Vegetables (but also meat) are also included in stews (for instance the Moroccan *tagine*, a pot with a characteristic tall conical lid, or the Northern African *dukkah*) in which are cooked slowly flavored with a large variety of herbs and spices, olive oil, possibly including dried fruits (i.e., raisins) and then served with couscous or Arab bread [178]. Seasoning is, however, a consistent characteristic of most Mediterranean recipes, in which herbs and spices are both cooked or used raw over both complex dishes or simple salads (often including tomatoes and olives) [35]. Several plant products are rich in heat-labile nutrients (including vitamin A, vitamin C, lycopene, and folates) that are preserved in uncooked vegetables; preparation of vegetables and cereals as soups and stews (which may also include legumes and meats) allows the preservation of the cooking medium with virtually no significant water-soluble nutrient loss from vegetables [179]. Some advantages can be also considered including meats, which are cooked at low temperatures for longer time, thus limiting the formation of mutagens/carcinogens generally produced with exposure to high temperatures [180]. A basic component of several dishes (especially used in Italian and Spanish first courses with pasta, rice, or bread) is the so-called *sofrito* (originally derived from the word “soffritto”, in Italian), a cooked mixture composed of extra virgin olive oil, tomatoes, onion and/or garlic, and various herbs and spices fried at lower temperature [181]; this technique allows the olive oil to retain potential beneficial properties, such as the (poly)phenol content [182].

Among protein sources, roasted meat and fish are not usually served with added sauces, dips, or gravies, which are not part of the Mediterranean culinary tradition, while toppings are mostly based on olive oil, vinegar, and herbs/seasonings and spices [183]. Baked meat and fish may be softened by a vegetable stock (including celery, carrots, onions, and fresh herbs) or soaked in milk (semi-skimmed) in substitution of other added animal fats, such as lard or bacon fat [184]. Legumes are generally boiled and included into salads often accompanied by whole grain cereals, such as the Lebanese *mujadara*, or mixed in stews, such as the Greek *fasolada*. Originally in Egypt, but now also spread in other Mediterranean areas, legumes are consumed as falafel, which are fried croquettes of bean or chickpea flour (also known as *panelle* in the Sicilian region). Variants of classical hummus are also used as spreads all around the region.

Finally, although not referring to a specific cooking method, the consumption of fruit in the Mediterranean area is generally at the end of a meal, intended to serve as a dessert. Fruit is occasionally mixed in fruit salads, especially during the summer period and may represent a key ingredient for many other desserts (such as, the Italian fruit *gelato* or *granita*, which are some sort of less processed ice cream). However, the historical, yet culturally preserved method of consumption is also dried, alone served as a sweet at the end of a meal, or within the context of more elaborated cooked recipes [185].

### Fermented foods

Another feature of Mediterranean cuisine having its roots in ancient times is the consumption of fermented foods. The fermentation practices, consisting of the transformation and preservation of food using common microorganisms such as bacteria, yeast, and mold, date back to 10,000 BCE in North African countries [186]. The fermentation spontaneously occurring in the ideal Mediterranean climate mostly interested milk products and nowadays still commonly applied in the whole Mediterranean area to produce a great variety of yogurt and later buttermilk, kefir, sour cream, and cheeses [187]. The products are typically fermented by lactic acid bacteria (i.e., belonging to genera such as Lactobacillus, Leuconostoc, and Streptococcus) which rely on fermentable sugars for their metabolism and growth [188]. Such processes of lacto-fermentation lead to a variation of organoleptic properties and an improvement in the functionality of bioactive metabolites by the fermenting microorganisms, finally enhancing their nutritional and functional properties [189]. Moreover, recent evidence suggests that fermented products may play an underrated role in the gut microbiota, acting as probiotics promoting a healthy gut and beneficial stimuli to the local districts (such as liver health) and distant ones (i.e., through the gut-brain axis) [190].

Fermentation did not involve only milk-derived products but also other plant- and animal-based foods. Among the former, besides grapes, cereals, and other fruits for the production of wine, beer, and other fermented spirits, commonly consumed fermented “garden” vegetables include (table) olives, carrots, onions, cauliflower, cucumbers, peppers, radishes, and turnips. Such salt-fermented vegetables retain many phytochemical properties including organic acids, vitamins, trace elements, and their (poly)phenol profile while counteracting the effects of anti-nutritive compounds and improving the nutritional value of inedible substrates [191]. There are a variety of other specific and more complex products belonging to Mediterranean culture: for instance, fermented sourdough bread is made by the fermentation of dough using wild *Lactobacillaceae* and yeast that may decrease the amount of carbohydrates and non-digestible oligo- and polysaccharides while increasing the bacterial synthesis of certain amino acids and the bioavailability of mineral and vitamins resulting in a lower glycemic index and improved digestibility [192]. Another commonly used fermented product is vinegar, produced by a double fermentation (alcoholic and acetic fermentation) of fruits and vegetables containing sugar and starch that may reduce the content of non-nutrients, such as (poly)phenols, phytates, and tannins, or make them more digestible while increasing the absorption of minerals [177]. Among animal foods, fermented fish and meat are generally salt-fermented products that can be consumed as individual foods or processed into condiments and used to provide specific flavor, odor, and taste [193]. The fermentation processes may increase the content of bioactive peptides which are currently studied for their potential health benefits [194]. Examples of fermented meat products commonly consumed in the Mediterranean area include fermented dry sausages (such as, *sucuk*) and *pastrami*, traditional dry-cured semi-fermented meat combined with red pepper, paprika, ground fenugreek, and garlic originally from Turkey. Among the oldest fermented fish products, garum was a fish sauce made from preserved fish in ancient Rome, and nowadays renewed as fermented *colatura di alici* (anchovy sauce) alongside *bottarga* (fish roe) in Italy, and *lakerda* (bonito steaks) in Greece.

On the downside, proper preparation of many of the aforementioned fermented food products involves the use of high quantities of sodium, which represents a major limitation for its effects on human health [195]. In fact, the salt-fermented products need to be dry and sodium concentration higher than 20% of their total weight in order to prevent the growth of pathogenic and putrefactive microorganisms [196]. However, such concentrations have also been demonstrated to potentially affect the risk of both cardiovascular conditions and certain cancers (i.e., upper aero-digestive and stomach ones) [197]. Moreover, fermentation of animal products may promote the production of biogenic amines from free amino acids, which may exert biological functions (such as, regulation of blood pressure, growth and nerve conditions, immune development, and metabolic activities) that can exacerbate toward respiratory, cardiovascular, and nervous systems health conditions [198].

### Pattern of alcohol consumption

Wine consumption persisted over the centuries until the modern era. Contrary to the common opinion that the Mediterranean diet is characterized by moderate intake of red wine, also white wine has always been consumed in the Mediterranean area, while in recent years there has been a growth for beer production also in southern European countries. Moderate wine consumption is an integral part of a traditional Mediterranean Diet and was found to be a major contributor to its overall beneficial effect in large cohorts of people living in the Mediterranean basin [199, 200].

Alcohol is a known carcinogen and potential driver of other detrimental effects, so to be advised to be entirely excluded from the diet [201]. While the content of alcohol could be considered relatively low for both types of beverages, and the content in (poly)phenols possibly being in part responsible for the ascribed benefits of both wine and beer on CVD risk [202], what is truly characterizing the alcohol consumption in the context of the Mediterranean diet is the drinking pattern: in fact, both alcoholic beverages are generally consumed in moderation during meals. Interestingly, most studies exploring the effects of alcohol consumption on human health mainly used a quantitative approach not distinguishing the occasion (i.e., during meals *vs*. far from meals) and the pattern (i.e., frequent low doses *vs*. binge drinking). Large meta-analyses from observational studies confirm that moderate alcohol intake would provide a substantial reduction of CVD risk and all-cause mortality [203], while a possible increased risk even at low doses for breast, liver, and colon cancer might occur [201]. However, a later study has reported a J-shaped relationship between alcohol consumption and 23 different diseases, including cancer [204]. It is worth noting that most of the evidence has been evaluated on cohorts of population living outside the Mediterranean area with alcohol drinking features far from those characterizing the Mediterranean diet [205]. Future studies are required to better explore alcohol-drinking occasions and patterns to provide a strong scientific rationale for the recommendation of total abstention or, on the contrary, demonstrate certain potential benefits at moderate doses possibly consumed during meals.

### Local production and environment preservation

The recent “planetary diet” proposed by the EAT-Lancet Commission shares various similarities with the traditional Mediterranean diet [206]. As further proposed, an Italian version better emphasizing the (ideal or actual) Mediterranean dietary habits has been reported to have some slight differences, especially concerning the protein sources (higher in nuts and legumes, milk and derivatives, and cereal-based foods in the Italian diet) [207]. However, additional observations should be considered. The original nature of the foods characterizing the dietary habits of the populations historically living in the Mediterranean area was locally produced, scarcely chemically treated to enhance the production, and minimally processed to prolong its preservation [208]. The peculiar geographical location of the Mediterranean territory characterized by mild-to-warm weather, irregular terrain, and scarcity of water, obliged to flexibility in agricultural practices, with a preference for crop rotation and mixed cultures, with limited and abrupt spaces to be cultivated but also taking advantage of the biodiversity that the fertile soil had to offer [209]. Since early times, Mediterranean food production has been characterized by seasonality and local production taking advantage of biodiversity to ensure availability and enhance its quality over the whole year (i.e., blending procedures of flour, olives, etc.) [210].

Globalization, mass production and distribution moved the aforementioned paradigm toward an all-year product availability from worldwide origins [211]. Such a situation, aside from the hedonistic characteristics of flavor, implies an environmental impact unrelated to the type of product (plant *vs*. animal-based) that is currently scarcely measured in scientific literature [212]. Among plant-based productions, there is evidence that intensification of food products characteristic of the Mediterranean area (i.e., olives and olive oil) may have a detrimental impact on the environment [213]. Such negative environmental effects of production might be limited through the implementation of sustainable farming practices. Within productions, an environmental-friendly approach, controlled and inspired by older and traditional low-input procedures, might support maintaining natural and social values in the Mediterranean area food production [214]. Plant-derived foods produced through agro-ecological methods (i.e., free from chemical pesticides) would permit the selection of rich naturally-occurring antibacterial phytochemicals (such as polyphenols) and reduce human exposure to chemical products [215]. Moreover, certain plant foods, such as legumes, also naturally improve soil fertility by fixing atmospheric nitrogen, thus reducing dependence on artificial fertilizers [216]. Also, traditional olive-based agroforestry systems are able to preserve habitat diversity, landscape complexity, and soil health by maintaining stability of sloping areas [217].

Consumption of seasonal, local productions would also impact the environment by reducing the ecological impact of the transportations [218]. Seasonal and local food may also require less effort in terms of pesticides and alternative growing methods, thus also affecting the overall nutritional quality of the product [219]. Moreover, the inclusion in the traditional cuisine of local crops and indigenous plants (besides harvested ones) may help preserve the extraordinary biodiversity characterizing the Mediterranean basin [220]. In fact, such natural biodiversity strongly influences agricultural practices and food diversification and availability over the year, promoting dietary variety that fosters nutritional guidelines [221]. Finally, consumption of seasonal, local productions may encourage the growth of local economies and survival of small entrepreneurs, resulting in the promotion of local economic sustainability [222].

### Level of food processing

In recent years, aside from the nutritional quality of the foods included in the Mediterranean dietary pattern, a growing interest has also been paid to the level of food processing. Based on a recent classification, foods have been considered ultra-processed when undergoing extreme industrial transformation through substantial alteration of the food matrix and addition of ingredients (not naturally present in the original food) aimed to increase durability, but also aesthetics, texture, and palatability [223]. High consumption of ultra-processed foods (UPFs) has been lately investigated as a potential threat for human health. A summary of nationally representative surveys shows that higher intake of UPFs leads to a poorer diet quality from a nutritional point of view; in fact, the NOVA group 4 of UPFs include the whole variety of recreational energy-dense, nutrient-poor foods which are generally rich in sugars (added), sodium, trans-fatty acids, while poor in fiber, protein, and micronutrients in general [224]. However, additional alerts have been raised also over those industrial, mass-produced, ready-to-eat goods that generally substitute fresher versions (cereals, bread) or complex dishes that need to be cooked [225]. These products may be more in line with dietary recommendations and provide an adequate nutrient profile; however, their content of natural or artificial additives and preservatives has been pointed out as a health threat itself [226]. Current evidence suggests that higher consumption of UPF is associated with higher risk of obesity, cardio-metabolic disorders, mortality, and detrimental mental health outcomes [227]. Although concerning, some criticisms have been raised against the definition of UPF: poor clarity of parameters used for their identification as well as the extreme variety of foods included may lead to unreliable comparisons across different populations (due to their different biological composition and the impossibility to define which are the most represented components) [228].

The consumption of UPFs has been recorded to reach up to 80% of daily energy intake in the US, UK, Canada, and Australia, while current rates of consumption in Mediterranean countries have been estimated to be much lower (an average of about 20%) [229]. Interestingly, higher adherence to the Mediterranean diet assessed through classical methods has been associated with lower consumption of UPFs [230–233]. It is intuitive to consider the Mediterranean dietary pattern a traditional aspect of the cultural background and the daily lifestyle of individuals living in the Mediterranean area, including its peculiarities concerning the cooking methods, the preference for fresh, natural foods over pre-packaged ones, as well as the habit of passing down culinary traditions across generations: however, as a result of globalization and abandonment of traditional lifestyles, UPFs are an emerging reality also in Mediterranean countries [234]. Thus, their inclusion in the diet needs to be further investigated when studying the level of adherence to the Mediterranean diet, as traditional methods of measure do not allow identifying whether the foods consumed are in fact unprocessed, minimally/culinary processed, or rather heavily processed.

### Physical activity

The term “diet” is derived from the Greek word “dìaita”, which means “lifestyle”. When describing the Mediterranean diet, it is often forgotten that it should not only refer to the dietary habits, but also certain behaviors that accompany the overall daily lifestyle of a person. The adoption of the Mediterranean diet in the past included important aspects of the daily behaviors that should not be considered disentangled from the dietary habits, but rather constituent of an overall exposure to a healthy lifestyle. The early studies on Mediterranean diet conducted in the southern Italian participants during the ‘60 are known to have involved mostly farmers [235]. This crucial aspect should be taken into account when considering that half of daily energy intake was provided by carbohydrates (from whole-grain cereals and legumes), which would counterbalance the high energy expenditure derived from the high levels of physical activity. Just after a few decades apart, the overall dietary and lifestyle habits have dramatically changed in those very same southern Italian villages [236], and during the following and more recent years, there has been a growing abandonment of physical activity as part of labor or lifestyle (i.e., commuting) behavior in favor of motorized transport and only recreational alternatives (limited to health-conscious individuals).

Globally, the number of deaths due to low physical activity have been estimated to almost reach 1 million in 2019 [237]. Nonetheless, the benefits of adequate physical activity span from cardio-metabolic outcomes to mental health, leading to an overall prolonged life and fewer years of life lost due to disability [238]. However, comprehensive evidence of literature underlined how a combination of dietary and physical activity interventions would have a higher impact on health and body weight [239]. Furthermore, some studies suggested a joint synergistic effect on mortality risk of higher adherence to specifically Mediterranean diet and high physical activity addressing the current research gap regarding interaction analyses regarding these two variables [240].

### Circadian rhythm and sleeping patterns

In the context of healthy lifestyles and physical activity, a growing area of interest in nutritional sciences concerns the relationship between diet and brain health [241]. While mechanistic insights on how food affects the human brain (i.e., through the gut microbiota), concerns still remain regarding the specific clinical manifestation of such influence [242]. Current evidence suggests that diet and sleep quality are two interconnected dimensions that affect human health [243]. Irrespective of the mechanisms involved, dietary behaviors and lifestyle have dramatically changed over time leading to disruptive behaviors if compared to the traditional Mediterranean lifestyle [244]. When looking back at the southern Italian farmers studied by Ancel Keys, as well as more recent older islanders living in the Mediterranean area, one can expect that labor day would start early in the morning, the out-of-home day would be characterized by a frugal meal followed by a short rest at mid-day (possibly with a nap in the fields) and an early dinner time when the main meal would be consumed [245]. More recent studies showed that morning chronotype (defined as a natural inclination of the body to wake up and be most active during the earlier part of the day) may be associated with higher adherence to the Mediterranean diet [246]. Sleep hygiene, intermittent fasting, and time-restricted eating (alternating periods of fasting or reducing the daily window of time of eating) are also currently investigated nowadays as eating patterns potentially associated with human health [247]. Regular sleeping patterns in line with sunlight exposure and (eventually) napping time, together with fewer eating occasions are probably the most contrasting lifestyle features when comparing a traditional Mediterranean lifestyle with modern life behaviors [248]. Based on the circadian rhythm and our ancestral biology, the human body is probably better designed to respond to food intake restricted to smaller windows of time within the day, for instance when exposed to daylight [249]. There is evidence that such eating patterns may affect metabolic and neuronal network activity which, in turn, regulate a variety of functions in the human body [250]. The resulting effects have been related to lower risk of several non-communicable diseases, including CVD [251], metabolic risk factors [252], certain cancers (prevention and survival) [253], and affective and cognitive disorders [254].

Studies on sleep behaviors and adherence to the Mediterranean diet are growing but often do not consider both dietary and lifestyle dimensions as a whole exposure. Some studies investigated the role of time restricted eating (i.e., breakfast/dinner skippers) and during Ramadan (fasting from dawn to sunset) in the context of the Mediterranean diet showing substantial benefits toward metabolic health [255, 256]. In contrast, social jetlag (circadian desynchrony with external day) has been associated with lower diet quality and abandonment of traditional dietary patterns [257].

### Social and cultural values

Westernized societies have developed an alarming culture of increased takeaway and ready-to-eat food consumption, which not only has health consequences (disruption of circadian rhythms, sleep disorders, possibly social isolation, and depressive symptoms) but has also removed people from enjoying the whole process of growing, cooking, and enjoying good wholesome food together. Up to date, the role of social engagement in cooking and experiencing meals together is hardly investigated in the context of studies exploring the relation between the Mediterranean diet and health outcomes, undermining an important aspect that could play a role, if not crucial for mental health outcomes, at least supposedly important if not unmeasured. Cooking time and consuming meals in company represent moments of conviviality, communication, and social exchange [258]. With the increased personal, societal, and financial burden of chronic physical and mental illness, getting back to the basics by promoting cooking skills and family/group meals could be such a simple yet powerful and empowering approach to healthcare and prevention.

Frugality represents another crucial principle of the classical Mediterranean diet investigated in the’60 s somehow connected with social values as well. As mentioned above, eating occasions represent moments of gathering aimed to establish and maintain the social structure of a group (i.e., a family) or to bring together different people in light of hospitality for a moment of confrontation, discussion, and sharing [259]. In contrast to the social importance of the meal (generally identified as a unique occurrence during the day), the other eating occasions were in fact characterized by frugality and moderation [260]. Current trends seem to have overcome such a paradigm, with continuative snacking (“recreational eating”) with discretionary foods (i.e., during working hours at the office, at home alone during computer activities, etc.) and quick main meals while watching television with poor communication and discussion occasions [261]. Assessing a dietary pattern in such a context seems not to truly capture the social and cultural elements characterizing the original Mediterranean diet. The totality of dietary indices does not take into consideration the lifestyle associated with adherence to the Mediterranean diet, thus substantially missing the point of contextualizing dietary habits with cultural beliefs of a population.

## Conclusions

In conclusion, the Mediterranean food culture is embedded within the history of populations living in the region and expression of their lifestyles and traditions, religions, and cultural backgrounds. Such dietary pattern should not be considered a simple list of ingredients or recommendations to follow decontextualized from the historical background, food quality, and lifestyle habits associated with it. The study of the Mediterranean diet should go beyond the “dietary pattern” itself and should also consider the true nature of the lifestyle and interaction with the traditional cultural heritage belonging to the population living in the Mediterranean area. While it is understandable that a simplified approach was needed to explore the effects of a Mediterranean dietary pattern on human health and its possible application to non-Mediterranean populations, it is now quite reductive to stop at considering what has been studied and advertised up to date while leaving out several other aspects equally important. Taking for granted the effects of the Mediterranean diet on health, future research should consider the suggested underrated aspects often (if not always) poorly investigated and start putting the foundation for the variety of features and values to be potentially applied globally through the concept of a “*Planeterranean*” diet.

## Data Availability

Not applicable.

## Abbreviations

ALA: Alpha-linolenic acid
CVD: Cardiovascular disease
DHA: Docosahexaenoic acid
EPA: Eicosapentaenoic acid
HCA: Heterocyclic amines
HDL: High-density lipoprotein
LDL: Low-density lipoprotein
MUFA: Mono-unsaturated fatty acids
PREDIMED: *Prevención con Dieta Mediterránea*
PUFA: Poly-unsaturated fatty acids
TMAO: Trimethylamine *N*-oxide
UPF: Ultra-processed food
